# The anticancer effects of Metformin in the male germ tumor SEM-1 cell line are mediated by HMGA1

**DOI:** 10.3389/fendo.2022.1051988

**Published:** 2022-11-23

**Authors:** Alessandro Salatino, Maria Mirabelli, Eusebio Chiefari, Marta Greco, Anna Di Vito, Giuseppe Bonapace, Francesco S. Brunetti, Fabio Crocerossa, Alan L. Epstein, Daniela P. Foti, Antonio Brunetti

**Affiliations:** ^1^ Department of Health Sciences, University “Magna Græcia” of Catanzaro, Catanzaro, Italy; ^2^ Department of Experimental and Clinical Medicine, University “Magna Græcia” of Catanzaro, Catanzaro, Italy; ^3^ Department of Medical and Surgical Sciences, University “Magna Græcia” of Catanzaro, Catanzaro, Italy; ^4^ Department of Pathology, USC Keck School of Medicine, Los Angeles, CA, United States

**Keywords:** Metformin, germ cell tumors, IGFBP1, HMGA1, SEM-1 cells

## Abstract

**Introduction:**

Germ cell tumors (GCTs) are the most common type of cancer in young men. These tumors usually originate from the testis, but they can occasionally develop from extragonadal sites probably due to primordial germ cells (PGCs) migration errors. Cisplatin-based chemotherapy is usually effective for male GCTs, but the risk of toxicity is high and new therapeutic strategies are needed. Although Metformin (Met) has been widely studied as a potential cancer treatment over the past decades, there is limited evidence to support its use in treating male GCTs. Additionally, the mechanism by which it acts on tumor cells is still not entirely understood.

**Methods:**

SEM-1 cells, a newly established human cell line of extragonadal origin, were treated with Met. Cell viability was studied by MTT assay, while cell migration and invasion were studied by the wound healing assay and the transwell assay, respectively. The effect of Met on 3D spheroid formation was determined by seeding SEM-1 cells in appropriate cell suspension culture conditions, and cell cycle was characterized by flow cytometry. Factors involved in PGCs migration and GCT invasion, such as IGFBP1, IGF1R, MMP-11 and c-Kit, together with cyclin D1 (a key regulator of cell cycle progression), and the upstream factor, HMGA1, were determined by immunoblots.

**Results:**

Treatment of SEM-1 cells with Met resulted in a potent and dose-dependent reduction of cell proliferation, as evidenced by decreased nuclear abundance of cyclin D1 and cell cycle arrest in G1 phase. Also, Met prevented the formation of 3D spheroids, and blocked cell migration and invasion by reducing the expression of IGFBP1, IGF1R and MMP-11. Both, IGFBP1 and MMP-11 are under control of HMGA1, a chromatin-associated protein that is involved in the regulation of important oncogenic, metabolic and embryological processes. Intriguingly, an early reduction in the nuclear abundance of HMGA1 occurred in SEM-1 cells treated with Met.

**Conclusions:**

Our results document the antiproliferative and antimigratory effects of Met in SEM-1 cells, providing new insights into the potential treatments for male GCTs. The anticancer properties of Met in SEM-1 cells are likely related to its ability to interfere with HMGA1 and downstream targets, including cyclin D1, the IGFs system, and MMP-11.

## Introduction

Metformin (Met) is an oral antihyperglycemic agent belonging to the biguanide family of drugs. It represents a synthetic derivative of galegine (the active ingredient of *Galega Officinalis*) and one of the oldest and most prescribed medications for type 2 diabetes (T2D) mellitus (1–3). Its first successful application in T2D-focused clinical trials has been reported in 1957 (4). Since then, Met’s popularity has grown over the years, and it is now one of the most commonly used drugs in the management of T2D (4). In pharmacodynamic studies, Met has been shown to improve whole-body insulin sensitivity, which help reduce blood glucose levels in patients with T2D and other insulin resistant states (5). However, despite more than 50 years of basic research efforts and clinical investigation, the specific molecular mechanism(s) by which Met exerts its peripheral insulin-sensitizing effects remains unknown. Some studies have informed that Met, by interfering with the mitochondrial bioenergetics, increases the cellular AMP/ATP ratio, leading to the activation of the AMP-activated protein kinase (AMPK) (6), a cytosolic metabolic sensor with a key role in the transcriptional repression of gluconeogenic target genes (6). More recently, other works, including our own, have highlighted a direct modulatory role of Met in nuclear events that regulate the function of chromatin-associated proteins and transcription factors, such as the hypoxia-inducible factor 1α (HIF-1α), which is known to modulate the transcription of hypoxia-related genes (7).

Despite uncertainties regarding its exact mechanism of action, as for other herbal derivatives (8–10), in recent years, Met has been investigated in other clinical conditions besides T2D, due to its wide availability and affordability (7). For example, Met is used to ameliorate insulin resistance and improve reproductive outcomes in women with polycystic ovary syndrome, and, although some controversies still exist, it is also used in the treatment of gestational diabetes as a pharmacological alternative to insulin (11). In addition, several epidemiological studies have shown that women with T2D treated with Met had lower rates of gynecological cancers (i.e., ovarian neoplasms) and a better prognosis than women treated with other antidiabetic agents (7). Despite these findings, little is known about the potential significance of Met in andrological tumors, such as prostate cancer and testicular cancer, for which the relationship with T2D is still debated (7).

The overall global incidence of male germ cell tumors (GCTs) is relatively low, with an estimate of about 75,000 cases in 2020 (12). Nonetheless, GCTs are the leading cause of cancer among men aged 30-35 years, when the age-specific incidence rate of these tumors reaches its peak level (12). There is currently a lot of interest in male GCTs, not only because of the novel epidemiological trends, but also because they share biological similarities with the embryonic germline and may be seen as developmental disorders (13). The last decade has seen a steady increase in the incidence of GCTs, which are often diagnosed in adolescents and young men who end up losing many years of healthy life (14). Fortunately, male GCTs usually respond well to cisplatin-based chemotherapy and cancer-specific deaths are uncommon occurrences (15). However, there are concerns about infertility and long-lasting treatment-related side effects, such as the appearance of second tumors and cardiovascular disease in patients with GCTs who receive cisplatin-based chemotherapy (16). Therefore, research into alternative treatment strategies is needed (15). The majority of male GCTs (approx. 90%) occur in the testis, while dysgenetic gonads and extragonadal sites account for the remaining 10% of cases (13, 14). Extragonadal sites are restricted to midline structures, such as the sacrococcygeal region, the retroperitoneal space, mediastinum, cervical and intracranial regions, all located along the migratory route of embryonic primordial germ cells (PGCs) towards the developing gonads (17). Migrating PGCs that do not properly reach the gonads can lead to extragonadal GCTs and impairments in sexual development and male fertility (13, 17–19).

The human extragonadal SEM-1 cell line, which was derived from the mediastinum of a patient diagnosed with an aggressive germ cell tumor, has recently been established (20). By taking advantage of this newly established model for studying the biology of male GCTs, herein we provide detailed *in vitro* evidence for a potent antiproliferative/antimigratory effect of Met in male GCTs. Furthermore, for the first time, we show that the anticancer effect of Met in these tumors is mediated by early downregulation of the nuclear high-mobility group A1 (HMGA1) protein, that is known to modify the architecture of chromatin and participate in the assembly of multiprotein DNA complexes that regulate gene transcription (21). Even more relevant, overexpression of HMGA1 has been indicated as a key player in male GCTs tumorigenesis (22).

## Materials and methods

### Cell culture and Met treatment

Early passage SEM-1 cells were grown and maintained as monolayers in T75 cell culture flasks containing RPMI-1640 culture medium supplemented with 10% (v/v) fetal bovine serum (FBS), 2 mM L-glutamine, and 1 mg/mL penicillin/streptomycin (Sigma-Aldrich, St. Louis, MO, USA), in a 37°C, humified 5% CO_2_ incubator. Before every experiment, cultured SEM-1 cells were synchronized *via* serum starvation (23) with FBS-free RPMI-1640 medium for 12 h overnight, thereafter resupplied with 10% FBS. Met, purchased as a highly soluble 1,1-dimethylbiguanide hydrochloride powder formulation (Cod.1115-70-4, Lexarna, Vanganelska, Sl), was dissolved in phosphate-buffered saline (PBS). On the day of experiment, a 100 mM stock solution of Met was made fresh and subsequently diluted in RPMI-1640 culture medium to reach the desired final treatment concentrations. To capture random biological variations, each experiment was performed at least three times.

### Evaluation of cell viability by tetrazolium reduction assay

The dose-dependent cytotoxic effects of Met on SEM-1 cells were assessed using the 3-[4,5-dimethylthiaoly]-2,5-diphenyltetrazolium bromide (MTT) cell viability assay, according to previously reported protocols (24). Briefly, 1 x 10^4^ SEM-1 cells were seeded into a 96-well plate and serum starved for 12 h overnight to synchronize the cell cycle. Then, complete RPMI-1640 culture medium was resupplied, and cells were treated with increasing Met concentrations (0.5 mM, 1 mM, 2.5 mM, 5mM, 10 mM) for 24 h. After this incubation period, 20 µl of the stock MTT solution, prepared beforehand by dissolving MTT-tetrazolium salts (Sigma-Aldrich, St. Louis, MO, USA) in sterile PBS, was added to each well, reaching a final MTT-tetrazolium concentration of 0.5 mg/mL, maintaining cells at 37°C in a humified atmosphere with 5% CO_2_. Two hours later, purple MTT-formazan precipitates were dissolved in pure isopropanol, and the absorbance (proportional to the number of viable cells in each well) read at 570 nm on the iMark Microplate Reader (Bio-Rad Laboratories Inc., Hercules, CA, USA). A set of wells without cells (complete RPMI-1640 medium only) containing the diluted MTT solution was used as blank for proper background subtraction. Untreated SEM-1 cells were used as negative controls and represented 100% of cell viability in each experiment.

### Evaluation of cell growth by cell counting

The trypan blue dye exclusion assay showing the live (unstained) and dead (blue) cells was used to measure cell growth over a period of up to 48 h, as it takes about two days for the SEM-1 cells to double their number in monolayer cultures (20). For this experiment, 5 x 10^4^ SEM-1 cells were seeded into 60 mm Petri dishes (P60) and allowed to grow in complete RPMI-1640 medium either in the presence or absence of Met (1 mM and 5 mM). At 24 h and 48 h timepoints, SEM-1 cells were detached from the plate by trypsinization and counted in a Burker’s chamber. Nonviable, positively stained cells with trypan blue were excluded from counting (25).

### Effects of Met on the generation of three-dimensional (3D) spheroids

In order to better explore the potential inhibitory effects of Met on the formation and aggressiveness of male GTCs, 3D spheroids of SEM-1 cells were established in adjunct to conventional monolayer culture systems (26). To generate 3D spheroids, 5 x 10^4^ SEM-1 cells were seeded into dedicated Ultra-Low attachment P60 culture dishes (Corning Inc., NY, USA), coated with an inert polymer hydrogel to prevent cell adhesion, and cultured in FBS-free RPMI-1640 medium, which is known to induce self-assembling spheroid formation in the presence of tumor cells with stem cell-like properties (27). Serum-free culture medium was changed every two days, either in the presence or absence of Met (1 mM and 5 mM), for up to 14 days. After this time period, the generated 3D spheroids were observed and characterized by digital inverted microscope imaging (EVOS FL Auto Imaging System, Life Technologies Corp., Carlsbad, CA, USA).

### Effects of Met on cell migration and invasion

The effects of Met on cell migration and invasion were respectively assessed by the wound healing assay and the transwell assay, as described elsewhere (15, 24). In wound healing assays, SEM-1 cells were plated into P60 culture dishes with complete RPMI-1640 medium and grown to 90-95% confluence. The scratch (or “wound”) was made by manually scraping the cell monolayer with a sterile p200 pipette tip. Then the medium was changed, and cells were grown either in the presence or in the absence of Met 5 mM for up to 24 h. To quantify wound closure over time, the images of the wound area, captured by digital inverted microscope imaging at times 0, 12 and 24 h after the scratch, were processed using ImageJ software and the Montpellier Resources Imagerie wound healing tool plugin (28). In transwell assays, 1 × 10^4^ SEM-1 cells were seeded into 24-well Costar Transwell ^®^ cell culture inserts (4 µm pore size; Corning Inc. NY, USA), in the presence or the absence of 5 mM Met, under low serum conditions (1% FBS). The outer well was filled with 800 µL of complete RPMI-1640 medium supplemented with 10% FBS, as the source of chemoattractant factors (i.e., IGFs). After incubation for 24 h, the SEM-1 cells that could migrate and adhere on the outer well plate were fixed in 10% formalin and stained with 4′,6-diamidino-2-phenylindole (DAPI, 1:500 Life Technologies Corp., Carlsbad, CA). Images were acquired by fluorescent microscopy on the FLoid Cell Imaging Station (Thermo Fisher Scientific Inc. Waltham, MA, USA). Invasion was determined by counting cells in five microscopic fields per well, and the average count was used to calculate the extent of invasion.

### Protein extraction and immunoblot analysis

Following the wound healing assay, total protein extracts were obtained from SEM-1 cells, according to standard procedures (29, 30). After 24 h of treatment, cells were detached from the plate by trypsinization, washed in cold PBS and solubilized in Nonidet P-40 (Sigma-Aldrich, St. Louis, MO, USA) lysis buffer (137 mM NaCl, 20 mM Tris-HCl,1 mM MgCl_2_, 1 mM CaCl_2_, 2 mM EDTA, 1.5% Nonidet P-40, 1 mM PMSF, 5 mM protease inhibitor mix) in a microcentrifuge tube at 4°C. After incubation on rotating wheel (1 h) and microcentrifugation (20 min x 16,000 *g*), the supernatant was collected, and total proteins quantified spectrophotometrically using the Bradford dye-binding method (Bio-Rad Protein Assay Dye Reagent Concentrate, Bio-Rad Laboratories Inc., Hercules, CA, USA). To obtain fractionated nuclear and cytosolic protein extracts from time course experiments, the cell pellets were resuspended in a cytosolic buffer (10 mM HEPES pH 7.9, 10 mM KCl, 0.1 mM EDTA, 1 mM DTT, and 0.5 mM PMSF), incubated for 15 min at 4°C on rotating wheel and then microcentrifuged at 7,000 *g* for 5 min at 4°C. The resulting nuclear pellet was resuspended in nuclear buffer (20 mM HEPES pH 7.9, 0.4 M NaCl, 1 mM EDTA, 1 mM EGTA, 1 mM DTT, and 1 mM PMSF), and then incubated and microcentrifuged again. The supernatants represented the nuclear extracts (15). Fractionated protein extracts were also quantified using the Bradford dye-binding method. Once denatured, the samples were loaded on SDS-polyacrylamide gels, transferred to nitrocellulose membranes in regular Tris-glycine-methanol buffer, and then probed with antibodies directed against: HMGA1 (sc-393213, 1:1000, Santa Cruz Biotechnology); Cyclin D1 (sc-753, 1:1000, Santa Cruz Biotechnology), a key modulator of the G1-S phase transition (31); matrix metalloproteinase 11, MMP-11 (sc-8836-R, 1:1000, Santa Cruz Biotechnology); insulin-like growth factor binding protein 1, IGFBP1 (sc-4685, 1:1000, Santa Cruz Biotechnology); insulin-like growth factor 1 receptor, IGF1R β subunit (sc-713, 1:1000, Santa Cruz Biotechnology), and the cell-surface transmembrane receptor c-Kit (sc-5535, 1:1000, Santa Cruz Biotechnology). As internal controls, anti-α-Tubulin (2144, 1:1000, Cell Signaling) and anti-β-Actin (4967, 1:1000, Cell Signaling) antibodies were used for total lysates and nuclear ones, respectively, given that β-Actin is known to associate with chromatin-remodeling complexes in the nucleus (32). The antigen-antibody complex was detected through incubation of the membranes for 1 h at room temperature with peroxidase-coupled goat anti-mouse or anti-rabbit antibodies (Dako, Agilent Technologies, Santa Clara, CA, USA) and revealed using the fully automated Alliance Q9 chemiluminescence imaging system (UVItec Ltd., Cambridge, UK).

### Immunofluorescence analysis

For the immunofluorescence mapping of specific nuclear protein changes, 5 x 10^4^ SEM-1 cells were grown on a suitable, high-optical quality coverslip, and after serum starvation to synchronize the cell cycle, they were exposed to Met 5 mM for 12 h. Cells were then fixed in 10% formalin, permeabilized with 0,1% Triton X-100 (Sigma-Aldrich, St. Louis, MO, USA) and incubated overnight at 4°C with the primary anti-HMGA1 antibody diluted in BSA 1%. The antigen-antibody complexes were detected by incubation for 1 h at room temperature with a FITC-conjugated anti-mouse secondary antibody diluted in BSA 1%. Nuclear counterstaining was carried out with DAPI according to manufacturer instructions. Coverslips were mounted on glass microscope slides using the ProLong Gold mounting solution (Thermo Fisher Scientific Inc. Waltham, MA, USA), and the images were acquired through confocal laser scanning microscopy on the Leica DMIRB/TC-SP2 system (Leica Microsystems GmbH, Wetzlar, DE).

### Cell cycle analysis

Met-induced variations in the cell cycle profile were analyzed by propidium iodide (PI) staining and flow cytometry on the BD FACSCanto II system (BD Biosciences, Heidelberg, DE). For this experiment, 1 x 10^6^ serum-starved, synchronized SEM-1 cells were exposed to Met 5 mM for 24 h and then fixed with 70% ethanol in PBS at -20°C overnight. On the day of acquisition, cells were washed in PBS three times and then incubated for 30 min at 37°C with 0.5 mL of PI staining solution, obtained by dissolving 5 μL of PI (initial concentration 50 μg/mL) and 6 μL of RNase A (initial concentration 100 μg/mL) in PBS. The flow rate (the number of cells detected) was adjusted to 10,000 events per second, and the different cell cycle phases were resolved by DNA content using the FACSDiva software v. 6.1.3 (BD Biosciences, Heidelberg, DE). Cells unexposed to Met were used as a control.

### Statistical analysis

Differences between unpaired data were analyzed using the Student’s t-test for the continuous variables and the Chi-square test for the proportions (GraphPad Software, Inc., San Diego, CA, USA). Test results with a *p* value < 0.05 were considered as statistically significant.

## Results

### Met suppresses cell viability and growth of SEM-1 cells

**Figure 1:**
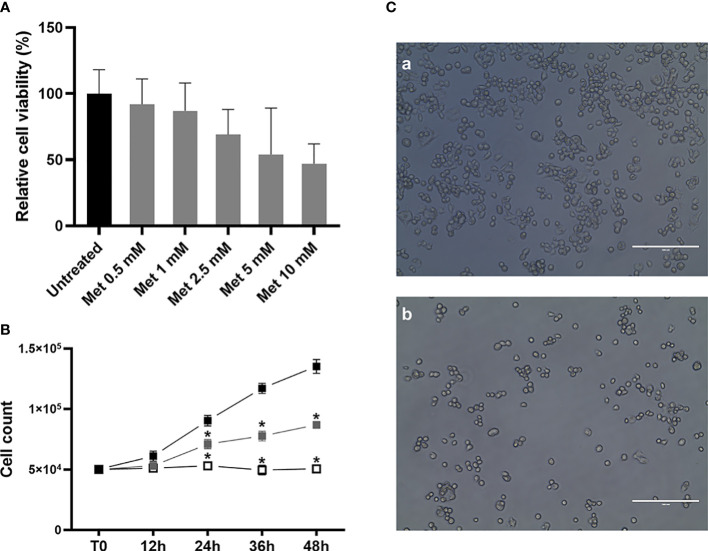
**(A)** MTT assay was performed to determine the IC_50_ value and cell viability after 24 h treatment with metformin (Met), at increasing concentrations (from 0.5 mM to 10 mM). Cell viability is the quantification of live cells and is expressed as the relative percentage to untreated controls. IC_50_ value (that is, the concentration of Met which exhibited 50% cell viability in SEM-1 cells) was 5 mM. Histograms are representative of a minimum of three replicates. Cell viability data are shown as mean + s.e.m. **(B)** Growth curves of SEM-1 cells, either untreated (black squares) or exposed to Met 1 mM (gray squares) and 5 mM (white squares) for up to 48 h, as obtained by the Trypan Blue dye exclusion counting method. *p < 0.05 *vs* untreated cells. **(C)** Representative pictures of SEM-1 cells in adherent culture conditions: a) untreated; b) exposed to Met 5 mM for 24 h. Scale bar: 200 µm.

To assess the antiproliferative effects of Met treatment on this *in vitro* model of male GCTs, and in order to identify the optimal concentration to be used in subsequent studies, we initially performed MTT cell viability assays on SEM-1 cells treated for 24 h with increasing doses of Met. As shown in **Figure 1**, exposure to increasing concentrations of Met was associated with a progressively enhanced suppression of cell viability, with a calculated absolute (or growth) half-maximum inhibitory concentration (IC_50_), i.e., the concentration of the tested drug inhibiting, *in vitro*, cancer cell proliferation by 50% (33) equal to 5 mM, indicating a potentially greater sensitivity of SEM-1 cells to Met with respect to other tumor cell lines (34). Cell growth curves using the trypan blue dye exclusion assay, in the presence of Met concentrations equal or lower than the IC_50_ value, confirmed the results from the MTT assay, indicating a 50% suppression of SEM-1 cell proliferation following a longer-term exposure (48 h) to a treatment dose as low as of 1 mM Met (i.e., the cutoff defining a supra-pharmacological dose) (35).

### Met inhibits the formation of 3D spheroids of SEM-1 cells

**Figure 2:**
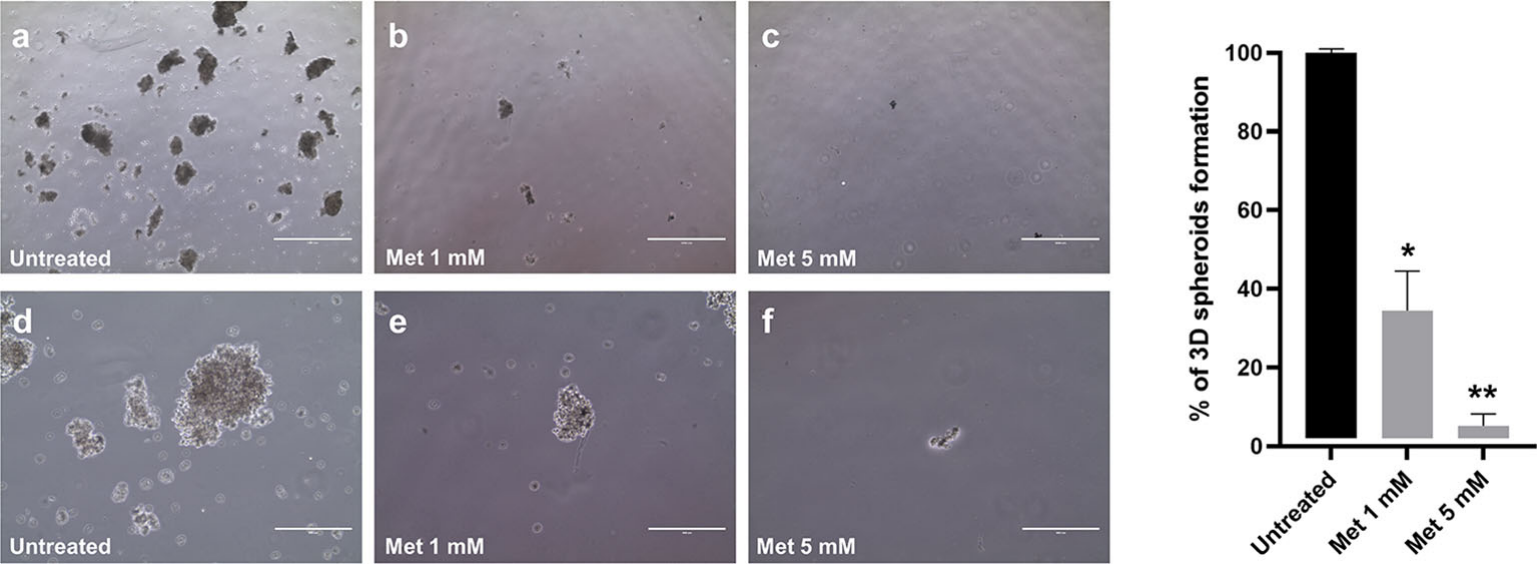
Dose-dependent effect of Met on 3D spheroid formation. Adherent SEM-1 cells were passaged to serum-free suspension media and tested for their ability to form 3D spheroids. Representative pictures: **(A, D)** untreated cells; **(B, E)** cells exposed to Met 1 mM for 14 days; **(C, F)** cells exposed to Met 5 mM for 14 days. Scale bars: 1000 µm **(A-C)** and 400 µm **(D-F)**. Histograms are representative of three replicates and express the mean 3D spheroids number (diameter > 50 µm) divided by the number of cells seeded relative to untreated controls. *p < 0.05 *vs* untreated cells (black bar), **p < 0.05 *vs* cells treated with 1 mM Met (grey bar).

Compared to adherent cultures, 3D spheroids are enriched with cells possessing stem-like properties that correlate with tumor formation and invasiveness (26, 27). Little is known about the effects of Met on 3D tumor spheroid growth (36). For the first time in this work, we addressed this issue in SEM-1 cells, which have been reported to express high levels of stemness-related markers (20) and, thus, are potentially capable to generate and tolerate long-term cultivation of 3D spheroids (37). As shown in **Figure 2**, we were successful in establishing 3D spheroids after passaging adherent SEM-1 cells to serum-free suspension media, indicating that an enrichment in cells with stem cell-related characteristics occurred. Met treatment resulted in a dose-dependent reduction in 3D spheroid number and size, with an almost complete loss of the ability to generate and maintain 3D spheroids in the culture medium.

### Met suppresses SEM-1 cell migration and invasion by affecting the IGFBP1/MMP-11 pathway

**Figure 3:**
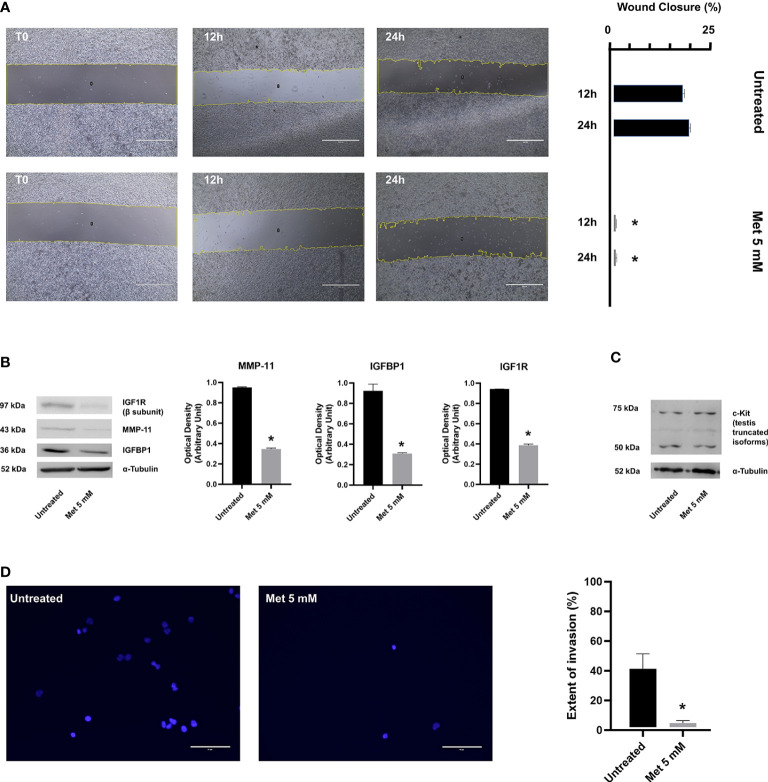
**(A)** Wound healing assay was performed to determine the migratory ability of SEM-1 cells during 24 h treatment with 5 mM Met. Wound closure was quantified as the relative percentage reduction of the wound area to time 0 (T0) at 12 h and 24 h of Met treatment, using the Image J software. Scale bar: 1000 µm. Histograms are representative of three replicates. *p < 0.05 *vs* untreated cells (black bars) at each time point. **(B)** Representative immunoblots of protein factors MMP-11, IGFBP1, IGF1R. Expression levels were normalized to α-Tubulin and quantified as relative arbitrary units. *p < 0.05 *vs* untreated cells (black bars). **(C)** A representative immunoblot of truncated c-Kit isoforms is shown. **(D)** Transwell assay was performed to determine the invasion ability of SEM-1 cells during 24 h treatment with 5 mM Met. Extent of invasion was determined as the proportion of seeded cells that adhered to the bottom well plate. The average cell count in five microscopic fields per well was performed using the Image J software. Scale bar: 75 µm. Differences between proportions were compared using the Chi-square test. *p < 0.05 *vs* untreated cells.

Cells in suspension culture have no migratory movement and currently there is a lack of accurate means for testing the effects of anticancer drugs on cell migration in 3D spheroids (38). At present, the most widely used method to evaluate and quantify cell migration is the wound healing assay, which is based on the mechanical creation of a scratch or “wound” (a cell-free area of approximately 1 mm in width) in a confluent cell monolayer (24). As shown in **Figure 3**, SEM-1 cells underwent this migration test either in the presence or absence of Met. The acquisition and quantification of wound area images at serial time points revealed a wound closure of 17% and 20%, respectively, at 12 h and 24 h after the scratch, in untreated SEM-1 cells; whereas cells exposed to 5 mM Met were unable to effectively migrate from the edges of the scratch and heal the wound. The reduction in cell migration by Met was dose-dependent, with a wound closure of only 1.9% and 8.7%, respectively, at 12 h and 24 h of treatment with 1 mM Met (data not shown). Also, the invasion capacity of SEM-1 cells was precluded in transwell assays, in which the proportion of transmigrated SEM-1 cells to outer well plates was significantly reduced by almost 10 times in the presence of 5 mM Met (**Figure 3**).

To corroborate these results, we investigated the effects of Met on some protein factors that are known to be involved in PGCs migration and metastatic capacity of extragonadal GCTs, such as IGFBP1 (39), IGF1R (39), MMP-11 (40) and c-Kit (41). IGFBP1 belongs to the IGFBP family, which is composed of six structurally similar proteins with high binding affinity for IGFs (IGFBP1 to IGFBP6). In contrast to other homologous proteins of the family, the expression of IGFBP1 is markedly altered by changes in the redox balance and cellular oxygen levels and responds to HIF-1α induction and suppression (42, 43), thereby raising the hypothesis that IGFBP1 might be a key determinant of Met anticancer activity. IGFBP1 not only regulates the bioavailability and activity of insulin-like stimuli during embryonic development and tumorigenesis, but has also its own biological activity, including the stimulation of tumor cell motility and adhesion, even in the absence of IGFs (44, 45). Following wound healing, a significant and marked decrease in IGFBP1 and IGF1R (the cognate receptor for unbound IGFs) protein expression was observed in SEM-1 cells treated with 5 mM Met compared to untreated cells (**Figure 3**). These changes in the expression of IGFBP1/IGF1R were associated with a significant reduction in MMP-11 protein abundance (39), suggesting that, when exposed to Met, SEM-1 cells may lose their motile and invasive capabilities through the downregulation of MMP-11 (**Figure 3**). Consistent with this, overexpression of MMP-11 appears to play a key role in cancer invasion and other directional migration processes (i.e., migration of PGCs to the developing gonads), although its precise role in male GCTs has not been yet clarified (39). Conversely, the abundance of truncated isoforms of c-Kit (a transmembrane protein receptor essential for germ cell survival, proliferation and migration), which are overexpressed in the majority of male GCTs (20), remained unchanged after treatment of SEM-1 cells with Met.

### Met induces HMGA1 protein depletion and arrests the cell cycle in G1 phase

**Figure 4:**
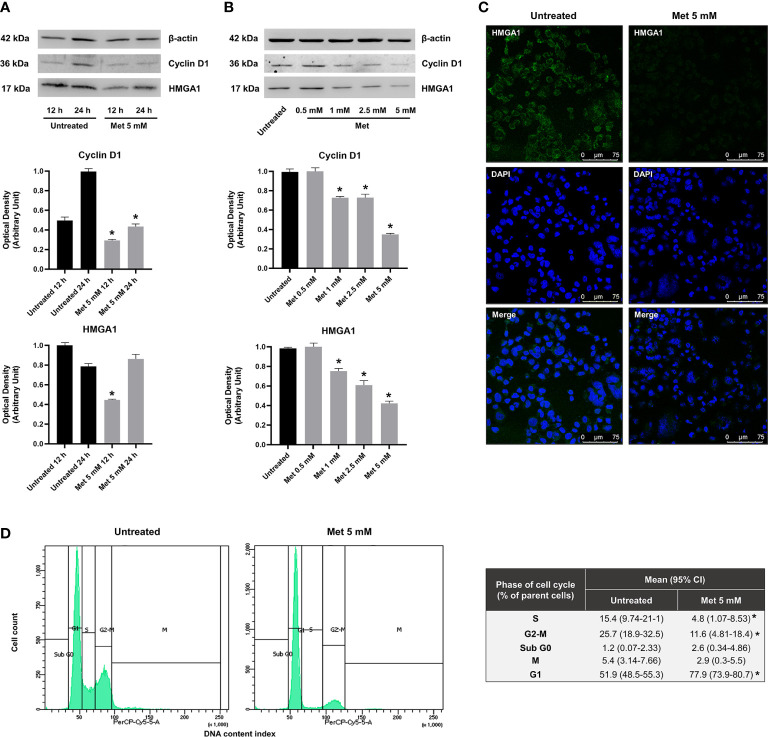
**(A)** Representative immunoblots of time-course experiments documenting a reduced expression of nuclear protein factors involved in cell cycle regulation (HMGA1 and cyclin D1) following 12 h and 24 h treatment with Met 5 mM in SEM-1 cells. *p < 0.05 *vs* untreated cells (black bars) at each time point. **(B)** Representative immunoblots documenting a dose-dependent reduced expression of HMGA1 and cyclin D1 following 12 h treatment with Met concentrations equal or lower than the IC_50_ (0.5 to 5 mM). *p < 0.05 *vs* untreated cells (black bars). **(C)** Immunofluorescence assay was performed to map HMGA1 (green fluorescence) in SEM-1 cells following 12 h treatment with Met 5 mM. Nuclear staining was performed with DAPI dye (blue fluorescence). Representative confocal images. Scale bar: 75 µm. **(D)** Distribution of cell cycle phases by PI staining in flow cytometry following 24 h treatment with Met 5 mM. Cell distribution across the phases of cell cycle was compared using the Chi-square test. *p < 0.05 *vs* untreated cells.

In non-GCT tumor types, there is evidence that MMP-11 expression positively correlates with HMGA1 protein production (46), an architectural chromatin-associated factor that induces stem cell-like properties, phenotypic reprogramming, invasiveness and spheroid-forming capacity in several cancer cell lines (47). Silencing of HMGA1 inhibits the formation of 3D spheroids, reduces the malignant features of breast cancer cells, and inhibits their migration and invasion both *in vitro* and *in vivo* (47, 48). These anticancer effects following HMGA1 silencing are consistent with what it is observed when treating SEM-1 cells with Met, thus supporting the hypothesis that HMGA1 may represent a molecular target for this drug. Interestingly, HMGA1 is necessary for the gene activation of IGFBP1 (49), whereas matrix metalloproteinases genes are all downstream targets of HMGA1 (50). To explore the possibility that HMGA1 could modulate the aforementioned effects of Met, we performed time-course experiments in synchronized SEM-1 cells treated with 5 mM Met. Under these conditions, Met caused a rapid depletion of the nuclear HMGA1 protein abundance, starting within 12 h after treatment. This depletion was gradually restored over the next 24 h, without reverting, however, the malignant phenotype of SEM-1 cells (i.e., spheroid-forming capacity, cell migration and proliferation). As shown in **Figure 4**, 12 h exposure of SEM-1 cells to increasing concentrations of Met led to a progressive decline in nuclear protein abundance of HMGA1. These results paralleled the decline in cell growth and proliferation as observed in the MTT assay, indicating that early inhibition of HMGA1 expression could be involved in the modulation of the cell cycle of SEM-1 cells by Met. Also, after 24 h of treatment with 5 mM Met, there was an increased proportion of SEM-1 cells in the G1 phase of the cell cycle (approximately +50% relative to untreated control cells), as evidenced by both the sustained loss in the nuclear accumulation of cyclin D1 and the results from the flow cytometry analysis (**Figure 4**). The inhibitory effects of Met on nuclear cyclin D1 accumulation were observed as early as at 12 h after treatment of SEM-1 cells with drug concentrations even lower than the IC_50_ value (**Figure 4**). This is in line with previous observations supporting the notion that, lowering HMGA1 protein abundance in pancreatic carcinoma cells that naturally overexpress HMGA1 and are highly responsive to insulin and IGFs, may impair the G1 phase progression by downregulating the protein expression of cyclin D1 (51).

## Discussion

Observational studies suggest that patients with T2D who take Met have a reduced risk of developing cancer and dying from cancer with respect to patients with T2D on other antidiabetic medications (7). Although the results from initial clinical trials failed to provide compelling data in non-diabetic cancer patients that underwent Met treatment (patients with early breast cancer and locally advanced non-small lung cancer) (52, 53), the anticancer potential of Met is underlined by various epidemiological and preclinical investigations. However, notwithstanding extensive research in this field, the cellular and molecular mechanisms underlying the anticancer effects of Met remain largely unknown, and do not appear to rely entirely on the classical activation of AMPK (35). Recently, we and others have shown that Met blocks HIF-1α expression in some human cell lines derived from aggressive forms of hepatocellular and prostate carcinomas, suggesting a direct involvement of this chromatin-associated protein in the anticancer activity of Met (7). HIF-1α is an essential modulator of cancer cell metabolism, as it orchestrates the dysfunctional adaptations of mitochondria to the hypoxic conditions of cancer (54). Another proposed anticancer mechanism of Met points to the drug-induced modulation of specific mitogenic stimuli, such as insulin, IGFs and related binding proteins, leading to decreased activation of insulin/IGF receptors, and thus, reduced proliferation and progression of tumors dependent on these signaling pathways (7). The IGFs system has an essential role in the normal development and embryonic migration of PGCs, as well as in the tumorigenic process of these cells, including cisplatin-resistance and metastatic spread of male GCTs, often characterized by sustained activity of the IGF1R signaling pathway (55). In this context, the potential of Met as an effective adjuvant therapy reverting cisplatin-resistance in male GCTs, has recently been reported (56). In particular, in the human TCam-2 cell line (a model of cisplatin resistant primary tumor of the testis), the antiproliferative and antimigratory activities of Met have been shown to intersect with the Hippo pathway (56), a newly discovered tumor suppressor cascade downstream of HMGA1, which is deregulated in several human cancers, including the malignant tumors of the breast (57). Herein, for the first time, we provide evidence that Met inhibits the proliferation, migration and *in vitro* spherogenic growth of SEM-1 cells in a dose-dependent manner. Furthermore, we show that Met interferes with signaling pathways that are under control of HMGA1, and that HMGA1 itself is an early target of Met.

HMGA1 is a small basic protein that binds to AT-rich chromatin domains and functions mainly as an architectural transcription factor by establishing a network of protein-protein and protein-DNA interactions (58). In previous works, we have reported that HMGA1 physically interacts with HIF-1α to fully activate the transcription of hypoxia-related genes relevant to tumorigenesis and glucose metabolism (59). In the nucleus, HMGA1 also structurally cooperates with the specificity protein 1 (Sp1) and the activator protein 2 (AP2) (60), whose overexpression is also a hallmark of male GCTs (21). Increased expression of HMGA1 has been associated with uncontrolled cell growth and proliferation, cell invasion, resistance to chemotherapy, maintenance of stem-like properties and metastatic spread in many human cancers, including male GCTs (61). Notably, HMGA1 is a positive regulator of the IGFs system, with its own DNA binding sites within the *IGFBP1* gene promoter (49), and it is also an inducer of matrix metalloproteinases and other genes involved in tumor invasion (50). In male GTCs, overexpression of HMGA1 has been attributed to the deregulation of miRNAs targeting the 3’ untranslated region of HMGA1 mRNA, such as mir-26a and Let-7 (23). Forced expression of these miRNAs has been shown to decrease HMGA1 protein levels and reduce motility in TCam-2 cells (62), supporting the idea that HMGA1 could be relevant for determining the aggressive traits of male GTCs. Suppression of HMGA1 has been proposed as a promising strategy for treating these tumors (61); however, the currently available HMGA1 inhibitors (i.e., distamycin polyamides) lack of specificity (63), while the potential interfering RNA molecules under development have not yet reached the clinical application stage (64). Studies on Met as an anticancer agent in male GCTs may help to overcome the problems of high-costs and long implementation time, two prerogatives of new drugs.

The hypothesis that suppression of HMGA1 is a determinant for the antiproliferative and antimigratory activities of Met comes from studies using cell lines of pancreatic carcinoma and nude mice xenografts (65). These studies found that two miRNA, mir-26a and Let-7, were significantly upregulated in Sw1990 and Panc-1 pancreatic cancer cells after 48 h Met treatment (65). Similar results were obtained in aggressive MDA-MB-231 breast cancer cells treated for 48 h with 10 mM Met (34). However, in SEM-1 cells, we observed that Met-induced HMGA1 downregulation occurred within the first 12 h of treatment with relatively low doses of Met, and that HMGA1 protein levels returned to normal afterwards. This finding, confirmed by us also in a cell line of hepatocellular carcinoma (unpublished data), raises the possibility that, in sensitive cancer cells, Met can influence the oncogenic functions of HMGA1 not only by means of miRNAs but also through means that are independent of miRNA-mediated regulation. The regulatory activities of HMGA1 on a variety of oncogenic, metabolic and embryological processes require a fine-tuned, spatial-temporal expression and they also depend on the post-translational modification status and relative DNA-binding affinity of this protein (22). Although the present work adds some pieces to the puzzle, the detailed mechanisms by which Met can influence HMGA1 biology are still not understood and further research is needed.

We are aware that the present study has some limitations, mostly related to the absence of non-morphometric characterization analysis of 3D spheroids and lack of a non-malignant control cell model for SEM-1 cells. However, in this cell model, which represents a unique tool for *in vitro* studies of male GTCs biology, our data indicate that Met exerts potent antiproliferative and antimigratory activities. These anticancer effects of Met appear to be mediated by HMGA1 and downstream targets, including cyclin D1, the IGFs system and MMP-11. Whether these Met effects can be successfully translated into clinical use for treating male GTCs is still premature, and it is unclear whether Met would also negatively affect the proliferation and migration of PGCs (and thus the pool of germ cells available for future spermatogenesis) in fetuses of women taking Met for the management of gestational diabetes. Met crosses the placenta (12), and male mice offspring exposed to Met *in utero* display reduced fertility and decreased spermatogenesis in adult life (66). Consistently, mice lacking HMGA1 display major histological and functional testis modifications, suggesting that this protein may play a vital role in normal sperm production and male gonadal development (67). As the results of this study are based on a male germ cell line of neoplastic origin, we cannot be sure how the potential HMGA1-mediated effects of Met in normal, non-malignant progenitor cells could be similar. Nonetheless, given the widespread use of Met, currently not limited to patients with T2D, we believe that the andrological implications of Met-induced modulation of HMGA1 should deserve attention.

## Data availability statement

The raw data supporting the conclusions of this article will be made available by the authors, without undue reservation.

## Author contributions

AS and MM (co-first authors) equally conceived the study, performed experiments, and drafted the manuscript. MG, AV and GB contributed to flow cytometry, immunofluorescence and immunoblot analyses. EC, FB, FC and DF provided valuable suggestions and contributed to data interpretation. AE contributed materials. AB supervised the study, contributed to data interpretation and edited the manuscript. All authors contributed to the article and approved the submitted version.

## Funding

The Article Processing Charge was funded by the Department of Health Sciences, University “Magna Græcia” of Catanzaro, Italy.

## Acknowledgments

Abstract of this work has been presented at the 2022 National Congress of the Italian Society of Pathology and Translational Medicine (SIPMeT), Ancona, 22-24 September 2022.

## Conflict of interest

The authors declare that the research was conducted in the absence of any commercial or financial relationships that could be construed as a potential conflict of interest.

## Publisher’s note

All claims expressed in this article are solely those of the authors and do not necessarily represent those of their affiliated organizations, or those of the publisher, the editors and the reviewers. Any product that may be evaluated in this article, or claim that may be made by its manufacturer, is not guaranteed or endorsed by the publisher.

## Abbreviations

AMPK, AMP-activated protein kinase; BSA, bovine serum albumin; DTT, dithiothreitol; EDTA, ethylenediaminetetraacetic acid; EGTA, ethylene glycol-bis-(beta-aminoethylether)-N,N,N’,N’-tetraacetic acid; FBS, fetal bovine serum; FITC, 5-fluorescein isothiocyanate; GCT, germ cell tumor; HEPES, N-2-hydroxyethylpiperazine-N-2-ethane sulfonic acid; HIF-1α, hypoxia-inducible factor 1α; HMGA1, high-mobility group A1; IC50, absolute half-maximum inhibitory concentration; IGF, insulin-like growth factor; IGFBP, insulin-like growth factor binding protein; IGF1R, insulin-like growth factor 1 receptor; Met, Metformin; MMP-11, matrix metalloproteinase 11; MTT, 3-[4,5-dimethylthiaoly]-2,5-diphenyltetrazolium bromide; PBS, phosphate-buffered saline; PGC, primordial germ cell; PI, propidium iodide; PMSF, phenylmethylsulfonyl fluoride; RPMI-1640, Roswell Park Memorial Institute 1640; T2D, type 2 diabetes
